# Optimization and Scale-Up of a Two-Level Electrodialysis Process for the Concentration of Lithium Chloride with High Energy Efficiency

**DOI:** 10.3390/membranes15090283

**Published:** 2025-09-22

**Authors:** Yu Zhang, Jikuan Wang, Liangyu Yu, Jiangnan Shen

**Affiliations:** 1 College of Chemical Engineering, Zhejiang University of Technology, Hangzhou 310014, China202005690524@zjut.edu.cn (L.Y.); 2 State Key Laboratory of Advanced Separation Membrane Materials, Zhejiang University of Technology, Hangzhou 310014, China

**Keywords:** two-level ED, LiCl concentration, energy efficiency, water migration amount, scale-up experiment, economic analysis

## Abstract

Traditional thermal concentration processes for LiCl, such as multi-effect evaporation and mechanical vapor recompression (MVR), suffer from drawbacks including high energy consumption and severe equipment corrosion. However, electrodialysis (ED) technology offers several advantages in the concentration process, including high efficiency, energy conservation, selective separation, and the absence of phase-change requirements. This study presents an innovative two-level ED process for efficient LiCl concentration, addressing the limitations of conventional thermal methods. Through systematic small-scale and scale-up experiments, we developed an optimized process achieving exceptional performance. The system attained Li^+^ concentrations of 22.17 g/L in the concentrated solution and 21.17 g/L in the recycled dilute solution, while reducing residual Li^+^ in discharge water to just 1.08 g/L. Remarkably, the process demonstrated significant energy efficiency, with a total consumption of only 85.22 kWh/t LiCl and a minimal water migration amount of 4.21 L/(m^2^·h). Economic analysis revealed substantial cost savings of 14.66 USD/t LiCl compared to traditional evaporation methods. These findings establish ED as a technically and economically viable solution for industrial LiCl concentration, offering both high efficiency and environmental benefits.

## 1. Introduction

Following the discovery of lithium in 1817, research into LiCl gradually began. Initially, it gained prominence in the metallurgy sector, where it is used as a fluxing agent in aluminum refining [1,2]. Starting in the 1970s, with the emergence of the lithium-ion battery concept, LiCl became a key raw material for battery electrolytes due to its excellent ionic conductivity and stability [3,4,5], leading to a surge in demand. Concurrently, its therapeutic effects on manic disorders in the pharmaceutical industry were discovered [6,7], opening up new application directions. Today, with the global energy transition and the rapid development of the new energy industry, higher demands are placed on the performance and production of LiCl. Additionally, under environmental pressure, there is a growing need for the recycling of lithium resources [8], driving research to focus on green and efficient processes and the expansion of innovative applications.

Currently, LiCl concentration primarily relies on thermal processes such as multi-effect evaporation [9] and MVR [10], but these methods suffer from high energy consumption and severe equipment corrosion (LiCl has strong hygroscopic and corrosive properties). Additionally, crystallization methods are easily disrupted by coexisting salts [11], affecting product purity. Therefore, developing new low-energy concentration technologies has become a research priority.

Nanofiltration (NF) technology, as a highly efficient membrane separation method, demonstrates unique advantages in treating LiCl solutions, particularly excelling in selective separation and energy consumption control [12]. Its pore size lies between reverse osmosis and ultrafiltration, enabling efficient retention of multivalent ions at lower operating pressures while maintaining permeability for monovalent ions like LiCl. This allows effective removal of impure ions during LiCl concentration, enhancing product purity. The process is relatively energy efficient, operationally straightforward, and easily integrated into existing production workflows. However, NF for LiCl concentration also presents notable drawbacks. Primarily, the membrane material exhibits limited retention efficiency for monovalent ions, resulting in suboptimal concentration rates. Some LiCl inevitably permeates the membrane with the solvent, reducing recovery rates. Additionally, high-concentration LiCl solutions may induce membrane fouling or degradation issues, such as scaling or diminished chemical stability, thereby shortening membrane lifespan and increasing maintenance costs. Furthermore, the NF process demands stringent pretreatment of feed solutions, and concentration polarization may further diminish separation efficiency, posing long-term economic challenges during sustained operation.

ED is a separation method based on ion exchange membranes and direct current electric fields, offering unique advantages in solution concentration and desalination [13,14,15]. This technology utilizes the selective permeability characteristics of cation exchange membranes and anion exchange membranes to achieve the directed migration of target ions under the influence of an electric field, thereby achieving separation and concentration [16]. The most notable feature of ED is its ability to operate at ambient temperature and pressure, avoiding the high energy consumption associated with traditional thermal concentration methods [17]. Its modular design allows the system to be flexibly adjusted according to processing scale, and the operation process does not require the addition of chemical agents, highlighting its environmental friendliness.

In recent years, researchers have explored various applications of ED technology in different fields. For example, bipolar membrane electrodialysis (BMED) can simultaneously achieve salt removal and acid-base regeneration, converting neutral salts into corresponding acids and bases [18,19,20]. Selective electrodialysis leverages the differential permeation capabilities of different ions (such as monovalent and multivalent ions, cations and anions, or specific target ions) under an applied electric field to achieve efficient separation and purification of mixed ions [21,22]. Exchange electrodialysis (EDM) utilizes the selectivity of ion exchange membranes and the chemical action of exchange agents to prioritize the migration and enrichment of specific ions (such as lithium and rare earth metals) from complex systems while minimizing interference from impurity ions [23,24]. Reverse-polarity electrodialysis (EDR) reduces membrane fouling and scaling by periodically switching electrode polarity [25,26,27]. Traditional electrodialysis (ED) is widely applied in ion concentration and separation, attracting significant attention in wastewater treatment and seawater/brackish water desalination [28,29]. For example, Zhou et al. [30] utilized ED technology to concentrate Li_2_SO_4_ solutions, employing two-level ED and achieving a final Li_2_SO_4_ concentration as high as 17.4 wt%. Similarly, Yan et al. [31] utilized ED technology to concentrate ionic liquids. The results showed that the membrane type and operating voltage drop of the cross-membrane stack were optimized to CJMC/MA membrane and 10 V, respectively. Additionally, due to the high concentration ratio (4.5) and low energy consumption (9.46 kWh/m^3^), the concentration efficiency of a volume ratio of 1:8 outperformed the partial recirculation operation mode and achieved a lower water transmission rate (10.3%). Xing et al. [32] utilized ED technology for the concentration of LiCl salt solutions. Experimental results indicated that ED technology is highly effective for concentrating LiCl salt solutions in lithium adsorption processes, with the Li^+^ concentration in the concentrate solution reaching 18–20 g/L.

Based on these research findings, this study aims to assess the feasibility of efficiently concentrating LiCl solution using two-level ED in a scaling-up experiment, and to validate and optimize the process flow for a small-scale experiment through a scaling-up experiment. The study investigates the effects of different volume ratios at each level of concentration on concentration efficiency, energy consumption, and water migration amount, as well as the impact of different operating temperatures on Li^+^ and water migration amount. This study is the first to use a two-level ED process to concentrate a LiCl solution, with the dilute solution produced in each level being recycled and reused for further concentration, ultimately achieving discharge standards. The research results indicate that both the small-scale and scaling-up experiments met the expected experimental requirements. Furthermore, the optimized scaling-up experimental design not only resolved the issue of excessive water migration amount in the small-scale experiment but also demonstrated high efficiency and controllability, laying the foundation for future industrial applications.

## 2. Materials and Methods

### 2.1. Reagents and Materials

The key parameters of the LiCl raw material solution are summarized in Table 1. The lithium sulfate used for preparing the electrolyte solution is sourced from Sinopharm Chemical Reagent Company Limited, with a chemical purity grade of analytical grade. The CEM used in the ED stack is CMT (Zhejiang Baichen Low Carbon Technology Co., Ltd., Shaoxing, China), while the AEM is AMT (Zhejiang Baichen Low Carbon Technology Co., Ltd., China). The key parameters of the ion exchange membranes are summarized in Table 2.

### 2.2. Experimental Principles and Device

**Figure 1:**
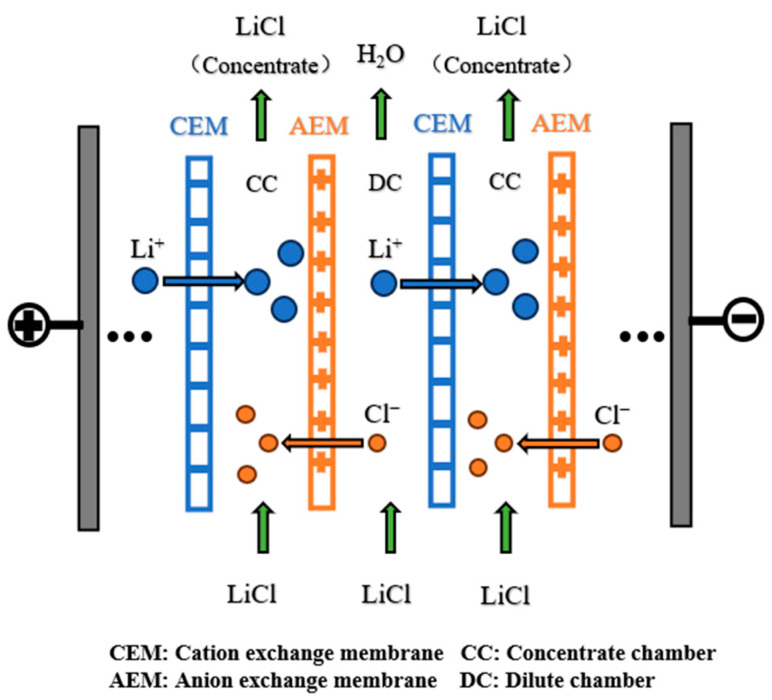
Schematic representation of the ED process.

Figure 1 illustrates the working principle of ED. In this experiment, a 0.3 mol/L Li_2_SO_4_ solution is added to the electrode chamber. Different volume ratios of the LiCl feed solution are added to the dilute chamber (DC) and concentrate chamber (CC), respectively. When a voltage is applied across the membrane stack, Li^+^ in the dilute compartment migrate toward the cathode, while Cl^−^ migrate toward the anode. Since the anion membrane only allows anions to pass through and the cation membrane only allows cations to pass through, ions accumulate in the concentrate compartment and are removed from the dilute compartment, thereby achieving the concentration of the target solution.

**Figure 2:**
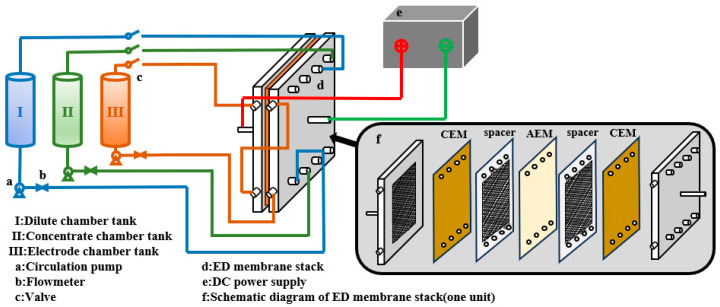
Diagram of the ED device.

Figure 2 shows the setup of the ED system. The solution flows through a closed-loop circuit consisting of a solution tank, a circulation pump, a flowmeter, an ED membrane stack, and a switch, before returning to the solution tank. A direct-current power supply is connected to the anode and cathode of the ED membrane stack, thereby generating the electric field required for ion transport. As shown in Figure 2, the ED membrane stack comprises an anode, a cathode, ion exchange membranes, and spacers. The anode and cathode are made of a ruthenium–titanium alloy. The ion exchange membrane used in the small-scale experiment has an effective area of 189 cm^2^, the spacers’ specification are 110 × 270 mm, and the stack consists of 10 ED units, including 11 CMTs and 10 AMTs. The ion exchange membrane used in the scaling-up experiment has an effective area of 4.275 m^2^, the spacers’ specification are 250 × 500 mm, and the stack consists of 60 ED units, including 61 CMTs and 60 AMTs.

**Figure 3:**
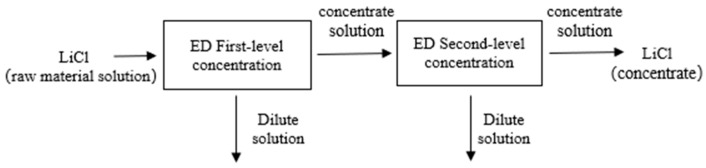
Small-scale experiment ED concentration process flow chart.

Figure 3 shows the process flow of the small-scale experiment for ED concentration. The main steps are divided into two parts: (1) the LiCl raw material solution is first subjected to first-level ED concentration; (2) the concentrate obtained from the first-level ED concentration of the LiCl raw material solution is then subjected to second-level ED concentration, and the concentrate obtained from the second-level ED concentration is the LiCl concentrate.

**Figure 4:**
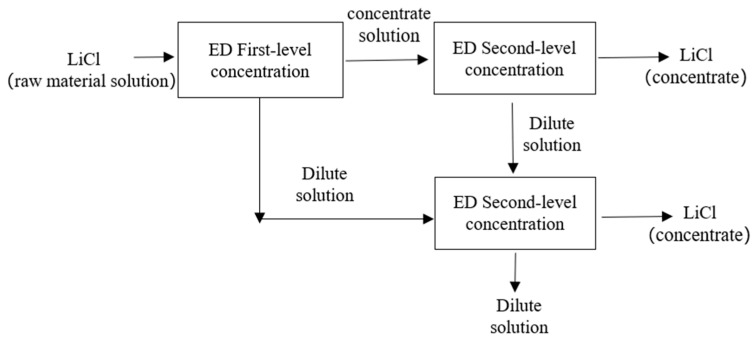
Scaling-up experiment ED concentration process optimization flow chart.

Figure 4 illustrates the optimized process flow for the scaling-up experiment ED concentration process. The LiCl concentration process can be divided into three steps: (1) First, the LiCl raw material solution undergoes primary ED concentration; (2) The concentrate obtained from the first-level ED concentration of the LiCl raw material solution is further subjected to second-level ED concentration. The concentrate obtained from the second-level ED concentration is the LiCl concentrate; (3) The dilute solution produced by the first-level and second-level ED is collected and subjected to a second-level ED concentration process. The resulting concentrate achieves the same concentration effect as in step (2), ensuring that the final freshwater meets discharge standards.

### 2.3. Experimental Procedure

#### 2.3.1. Selection of Voltage

Increasing the voltage appropriately can enhance the electric field strength and accelerate the migration rate of ions, thereby improving concentration efficiency [33]. However, when the voltage is too high, the system may reach or exceed the limiting current density, leading to concentration polarization, i.e., the formation of an ion depletion layer on the membrane surface, causing a sharp increase in resistance and energy consumption [34,35,36]. At this point the dissociation of water molecules (H^+^ and OH^−^ migration) replaces the migration of salt ions, reducing current efficiency and potentially causing local pH changes and membrane fouling [37] which affect membrane performance and lifespan. To determine the optimal operating parameters for the scaling-up experiment, it is determined that the ED first-level concentration is consistent with the experimental conditions of the small-scale experiment, and the feeding volume ratio of both the dilute solution tank and concentrate solution tank is 1:1, with a volume of 20 L. The voltage is set to 55, 60, and 65 V, respectively, and the effect of ED first-level concentration experimental process voltage on concentration efficiency, water migration amount, and energy consumption are investigated.

#### 2.3.2. Selection of Volume Ratio

In practical applications, the volume ratio between the DC and the CC need to be optimized according to specific application requirements. For applications requiring rapid production of high-concentration concentrate, the volume of the CC can be appropriately reduced, but this must be accompanied by the optimization of current density and flow rate to prevent membrane fouling. For systems requiring long-term stable operation, a larger CC volume is more suitable as it balances concentration efficiency with membrane lifespan [38,39]. Additionally, the selection of the volume ratio should consider the initial concentration of the feed solution, the target concentration multiple, and the overall energy efficiency of the system, with the optimal ratio determined through experimentation or simulation. To determine the optimal operating parameters for the scaling-up experiment, it is determined that the ED second-level concentration (concentrate solution) initial solution volume ratios between the dilute solution tank and the concentrate solution tank should be set to 15 L:3 L, 20 L:3 L, and 25 L:3 L. The ED second-level concentration (dilute solution) initial solution volume ratios between the dilute solution tank and the concentrate solution tank should be set to 25 L:3 L, 26 L:3 L, and 27 L:3 L. The effects of the initial solution volume ratio between the dilute solution tank and the concentrate solution tank on concentration efficiency, water migration amount, and energy consumption are investigated in the ED second-level concentration (concentrate solution) and ED second-level concentration (dilute solution) process.

#### 2.3.3. Selection of Temperature

The effect of temperature on the ED concentration process is significant, primarily manifesting in aspects such as ion migration, membrane performance, energy consumption, and system stability. Increasing temperature typically enhances ion migration rates and conductivity, thereby improving concentration efficiency and reducing energy consumption [40]. However, excessively high temperatures may cause membrane structural changes, reduced selectivity, and accelerated membrane fouling or scaling [41]. Conversely, low temperatures can slow ion migration rates, increase energy consumption, and even affect membrane mechanical strength [42]. Therefore, in practical operations, temperature optimization is necessary to balance efficiency, energy consumption, and equipment lifespan. To determine the optimal operating parameters for the scaling-up experiment, it is determined that the effect of the ED first-level concentration experimental process temperature on the Li^+^ and water migration amount are to be investigated. The reaction temperature control ranges are 25–27 °C, 27–29 °C, and 29–31 °C, respectively. The reaction temperature is displayed in real time by the temperature sensor on the electrodialysis equipment. This electrodialysis device is designed with an external circulation cooling pipeline to cool the circulating solution in each compartment. Only by introducing cooling water into the external circulation pipeline can the cooling effect be achieved. By controlling the flow rate of the cooling water, the reaction temperature can be further controlled within the required range.

With a flow rate of 1 m^3^/h for each chamber, record the conductivity and volume of the DC and CC every ten minutes. When the conductivity of the ED first-level concentration dilute solution side is less than 95 mS/cm, the experiment is stopped to prevent concentration polarization; when the conductivity of the ED second-level concentration dilute solution side is less than 20 mS/cm, the corresponding Li^+^ concentration is around 1 g/L and the experiment is stopped.

### 2.4. Analysis Method

1. Ion Concentration

Li^+^ concentration (m_1_, g/L) is calculated based on chloride ions in solution, which is as follows (Equation (1)):

(1) $$m_{1}=\frac{{M_{1}m}_{2}}{M_{2}}$$

where M_1_ represents molar mass of Li^+^ in the solution (g/mol), M_2_ represents molar mass of Cl^−^ in the solution (g/mol), and m_2_ represents Cl^−^ concentration in the solution.

2. Water Migration Amount

For the water migration amount (Q, L/(m^2^·h)) during ED, the volumes of dilute solution and concentrate solution are not constant, as water molecules migrate through the membrane along with ions. Therefore, the final assessment is based on the final volume of the CC, which is shown in the following Equation (2):

(2) $$Q=\frac{V_{2}-V_{1}}{S\cdot t}\times 100\%$$

where V_1_ represents initial volume of concentrate solution (L), V_2_ represents final volume of concentrate solution (L), S represents effective area of membrane (4.275 m^2^), and t represents total experiment runtime (h).

3. Ion Migration Amount

Li^+^ migration amount (N, g/(m^2^·h)) is calculated based on concentrate solution to calculate the migration amount of Li^+^. This is shown in Equation (3) as follows:

(3) $$N=\frac{C_{1}\cdot V_{1}-C_{2}\cdot V_{2}}{S\cdot t}$$

where C_1_ represents concentrate solution final concentration (g/L), V_1_ represents concentrate solution final volume (L), C_2_ represents concentrate solution initial concentration (g/L), V_2_ represents concentrate solution initial volume (L), S represents effective area of membrane (4.275 m^2^), and t represents total experiment runtime (h).

4. Energy Consumption

Energy consumption is calculated based on the electrical energy consumed throughout the entire ED process, which is as follows (Equation (4)):

(4) $$EC=\frac{\int_{0}^{t}U\cdot I\cdot dt}{M\cdot \left(C_{t}\cdot V_{t}-C_{0}\cdot V_{0}\right)}$$

where U is the voltage of the membrane stack at time t (V); I is the current of the membrane stack during the entire operation process (A); t is the operation time (min); M is the molar mass of lithium (g/mol); C_0_ and C_t_ are the concentration of the DC at time 0 and t (min), (mol/L); and V_0_ and V_t_ are the volumes of the DC at time 0 and t (min), (L).

## 3. Results and Discussion

### 3.1. The Results of the Small-Scale Experiment

**Figure 5:**
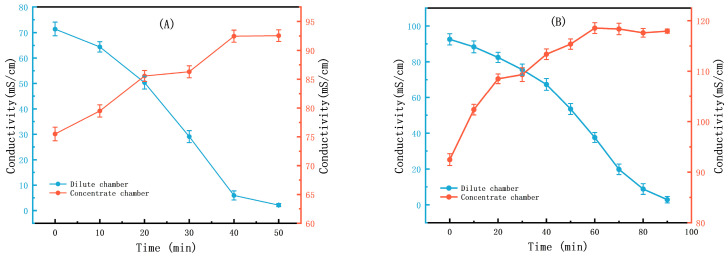
(**A**) Conductivity of the DC and CC ED first-level concentration; (**B**) conductivity of the DC and CC ED second-level concentration.

Figure 5A shows that in ED first-level concentration the feeding volume ratio of both the dilute solution tank and concentrate solution tank is 1:1, with a volume of 500 mL. After ED first-level concentration, the conductivity is concentrated from 75.8 (±2.04) mS/cm to 92.5 (±1.25) mS/cm. Figure 5B shows that in ED second-level concentration the feeding volume ratio of both the dilute solution tank and concentrate solution tank is 800 mL:200 mL. After ED second-level concentration, the conductivity is concentrated from 92.5 (±1.25) mS/cm to 117.6 (±0.15) mS/cm. The Li^+^ concentration on the concentration side is 24.77 g/L and the Li^+^ concentration on the dilute side is 1.121 g/L. The Li^+^ concentration effect of the small-scale trial is basically in line with expectations.

**Figure 6:**
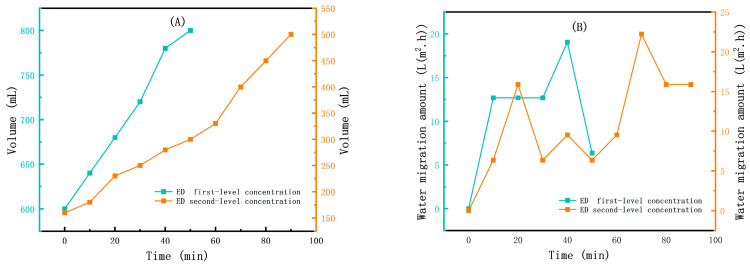
(**A**) CC volume of the ED first-level concentration and ED second-level concentration; (**B**) water migration amount of the ED first-level concentration and ED second-level concentration.

Figure 6A shows the ED first-level concentration and ED second-level concentration volume–time relationship chart. As time increases, the volume of the concentrate solution in the CC continues to rise, indicating that during the migration of ions a significant number of water molecules migrate to the CC, causing the volume of the CC to increase. Figure 6B shows the CC water migration amount of ED first-level concentration and ED second-level concentration. The water migration rate per unit of time in both processes is enormous, reaching a maximum of 22.22 L/(m^2^·h) and a minimum of 6.35 L/(m^2^·h). The total water migration amount during the process was 24.69 L/(m^2^·h). Excessive water migration can lead to increased process energy consumption and exacerbate the risk of membrane fouling and scaling [43]. Furthermore, the process did not consider the recycling of the dilute solution from the two-level ED, so the process is verified and optimized during the subsequent scaling-up experiment.

### 3.2. The Results of the Scaling-Up Experiment

#### 3.2.1. The Effect of Voltage on the ED Concentration Process

**Figure 7:**
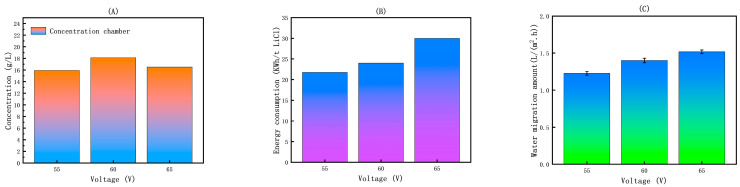
(**A**) Effect of voltage on the concentration of Li^+^ in ED first-level concentration; (**B**) effect of voltage on energy consumption in ED first-level concentration; (**C**) effect of voltage on water migration amount in ED first-level concentration.

Figure 7A illustrates that at a voltage of 60 V the maximum concentration of Li^+^ reaches 18.12 g/L, indicating that this voltage provides an optimal driving force for Li^+^ migration and concentration. Figure 7B illustrates that energy consumption exhibits a direct positive correlation with the applied voltage, as higher voltages increase current flow and consequently lead to greater ohmic losses across the membrane stack. Figure 7C illustrates that water migration in electrodialysis primarily occurs through electroosmotic drag accompanying ion transport, and increasing the voltage enhances both ion migration rates and the associated water movement [44].

Based on a comprehensive analysis of the voltage effects on concentration efficiency, energy consumption, and water migration amount, 60 V was selected as the optimal operating voltage for subsequent experiments. This determination reflects a balanced optimization among competing factors: while higher voltages enhance Li^+^ concentration they also result in disproportionate increases in energy consumption and undesirable water migration. The 60 V condition achieves a significant Li^+^ concentration (18.12 g/L) while maintaining acceptable energy efficiency and minimizing excessive water migration. This optimization approach aligns with established electrodialysis principles, where operational parameters are adjusted to maximize separation efficiency while minimizing energy consumption [45].

#### 3.2.2. The Effect of Initial Volume Ratio on the ED Concentration Process

The experimental results indicate that the initial volume ratio has a significant impact on the ED concentration process, which is primarily manifested in the following two aspects: changes in solution conductivity and water migration amount. This is because the initial volume ratio directly determines the initial ion concentration in the concentrate and dilute compartments, which in turn governs the system’s conductivity, the risk of concentration polarization, and the osmotic driving force [46].

**Figure 8:**
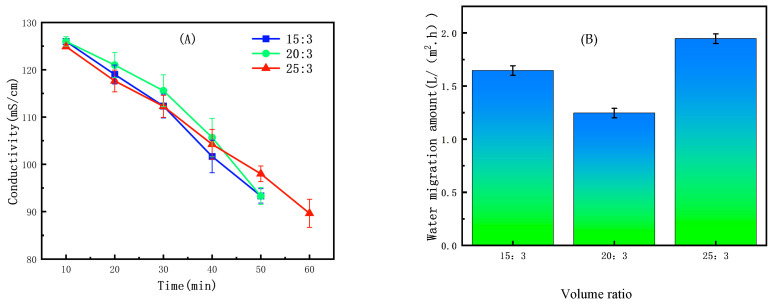
(**A**) Effect of initial volume ratio on conductivity in ED second-level concentration (concentrate solution). (**B**) Effect of initial volume ratio on water migration amount in ED second-level concentration (concentrate solution).

In ED second-level concentration (concentrate solution), when the initial volume ratio was set to 20:3 (Figure 8A) the water migration rate reached a minimum of 1.29 (±0.05) L/(m^2^·h) (Figure 8B). This optimal performance occurs because a lower volume ratio creates a higher initial ion concentration in the concentrate compartment. This results in higher conductivity and greater current efficiency, which enhances the driving force for ion migration [47]. Although this high concentration difference can promote osmotic water flux into the concentrate, the net benefit of improved electrical efficiency dominates at this ratio, minimizing the overall water migration amount.

**Figure 9:**
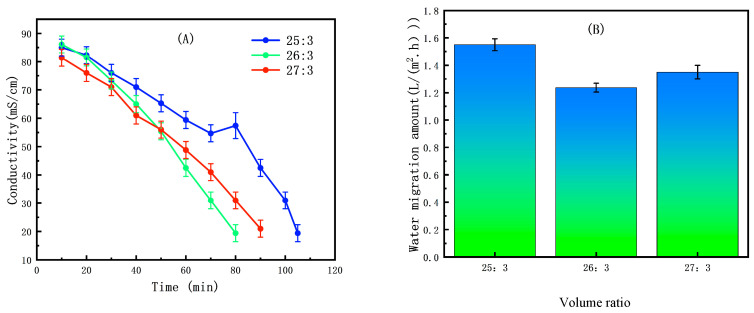
(**A**) Effect of initial volume ratio on conductivity in ED second-level concentration (dilute solution). (**B**) Effect of initial volume ratio on water migration amount in ED second-level concentration (dilute solution).

Conversely, in ED second-level concentration (dilute solution) the optimal initial volume ratio increased to 26:3 (Figure 9A), at which point the water migration rate further decreased to 1.26 (±0.04) L/(m^2^·h) (Figure 9B). The different requirement arises because the primary challenge in the dilute compartment is avoiding concentration polarization. A higher volume ratio means a larger reservoir and a lower initial ion concentration, which significantly delays the onset of polarization at the membrane surface. This maintains lower system resistance and stable, efficient operation, allowing for more effective ion removal and a further reduction in the water migration amount.

This indicates that different initial volume ratio optimization strategies are required for different solution systems; concentrated solutions are suitable for lower volume ratios (20:3) to maximize electrical migration efficiency, while dilute solutions perform better at higher volume ratios (26:3) to prioritize stability and minimize concentration polarization.

This finding has important implications for the optimization of ED processes. In practical applications, the appropriate initial volume ratio parameter should be selected based on the type of target solution to achieve optimal concentration efficiency and water migration control. The research results not only confirm the critical role of the initial volume ratio but also reveal the differences between concentrated solutions and dilute solutions under optimized conditions, providing a theoretical basis for the precise regulation of ED processes. Further studies could explore the synergistic effects of other operational parameters with the initial volume ratio to comprehensively enhance the overall performance of ED systems.

**Figure 10:**
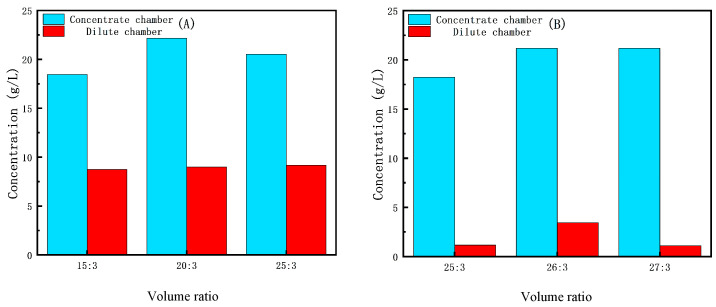
(**A**) Effect of initial volume ratio on the concentration of Li^+^ in ED second-level concentration (concentrate solution). (**B**) Effect of volume ratio on the concentration of Li^+^ in ED second-level concentration (dilute solution).

Figure 10A illustrates that in ED second-level concentration (concentrate solution) the final Li^+^ concentration can be increased by adjusting the volume ratio of feedstock between the DC and the CC. As shown in Figure 10A, when the volume ratio of feedstock between the DC and the CC is 15:3, which results in a Li^+^ concentration of 18.15 g/L, the final dilute solution Li^+^ concentration is 9.01 g/L. When the volume ratio of feedstock between the DC and the CC is 25:3, which results in a Li^+^ concentration of 20.53 g/L, the final dilute solution Li^+^ concentration is 9.24 g/L. The optimal effect is achieved when the volume ratio of feedstock between the DC and the CC is 20:3, resulting in a Li^+^ concentration of 22.17 g/L and a final dilute solution Li^+^ concentration of 9.17 g/L. Figure 10B illustrates that in ED second-level concentration (dilute solution) when the volume ratio of feedstock between the DC and the CC is 25:3, which results in a Li^+^ concentration of 18.23 g/L, the final dilute solution Li^+^ concentration is 1.16 g/L. When the volume ratio of feedstock between the DC and the CC is 26:3, which results in a Li^+^ concentration of 20.53 g/L, the final dilute solution Li^+^ concentration is 3.43 g/L. The optimal effect is achieved when the volume ratio of feedstock between the DC and the CC is 27:3, resulting in a concentrate solution where Li^+^ concentration is 21.17 g/L and a final dilute solution where Li^+^ concentration is 1.08 g/L, both of which meet the expected standards.

#### 3.2.3. The Effect of Temperature on the ED Concentration Process

**Figure 11:**
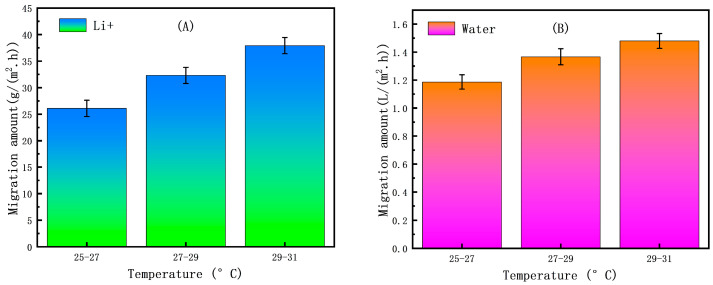
(**A**) Effect of temperature on Li^+^ migration amount. (**B**) Effect of temperature on water migration amount (taking the ED first-level concentration process as an example).

Figure 11 illustrates that within the appropriate temperature range (≤35 °C, avoiding high temperatures that could damage the membrane), under otherwise unchanged conditions, appropriately increasing the temperature of the ED concentration experiment can greatly increase the migration amount of Li^+^. This is because higher temperatures reduce the viscosity of the solution and increase the diffusion coefficient and migration rate of ions, and because higher temperatures accelerate the transport of ions through the ion exchange membrane. At the same time, faster ion migration indirectly drives more water molecules through the membrane because hydrated ions carry some water molecules with them as they migrate. However, within the appropriate temperature range, water migration amount can be maintained within an acceptable and controllable range. When the temperature range is 29–31 °C, the maximum Li^+^ migration amount is 38.26 (±2.52) g/(m^2^·h) and the water migration amount is 1.52 (±0.10) L/(m^2^·h).

#### 3.2.4. Analysis of Total Energy Consumption for Optimal Process Condition

The analysis of total energy consumption is one of the core objectives of the scaling-up experiment, providing key data support for the industrialization of LiCl solution concentration by ED technology. Therefore, this study combined the optimal process conditions with the other two conditions in a controlled experiment with the following conditions:

(1) The reaction temperature control ranges are 29–31 °C. The ED first-level concentration initial solution volume ratios between the dilute solution tank and the concentrate solution tank are set to 20 L:20 L. The ED second-level concentration (concentrate solution) initial solution volume ratios between the dilute solution tank and the concentrate solution tank are set to 20 L:3 L. The ED second-level concentration (dilute solution) initial solution volume ratios between the dilute solution tank and the concentrate solution tank are set to 27 L:3 L.
(2) The reaction temperature control ranges are 27–29 °C. The ED first-level concentration initial solution volume ratios between the dilute solution tank and the concentrate solution tank are set to 20 L:20 L. The ED second-level concentration (concentrate solution) initial solution volume ratios between the dilute solution tank and the concentrate solution tank are set to 15 L:3 L. The ED second-level concentration (dilute solution) initial solution volume ratios between the dilute solution tank and the concentrate solution tank are set to 25 L:3 L.
(3) The reaction temperature control ranges are 25–27 °C. The ED first-level concentration initial solution volume ratios between the dilute solution tank and the concentrate solution tank are set to 20 L:20 L. The ED second-level concentration (concentrate solution) initial solution volume ratios between the dilute solution tank and the concentrate solution tank are set to 25 L:3 L. The ED second-level concentration (dilute solution) initial solution volume ratios between the dilute solution tank and the concentrate solution tank are set to 26 L:3 L.

**Figure 12:**
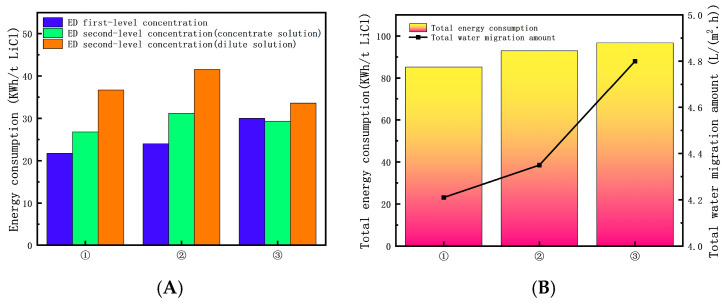
(**A**) Energy consumption under different experimental conditions. (**B**) Relationship between total energy consumption and total water migration amount.

Figure 12A illustrates the total energy consumption values of the comprehensive experimental process after comparing three different process conditions. (1) is the optimal process condition, with the lowest total energy consumption of 85.22 KWh/t LiCl. Figure 12B illustrates that the greater the total energy consumption of the process, the greater the total water migration amount. The reason for this is that more water molecules need to move, which takes energy to overcome the membrane resistance. Therefore, appropriately reducing the total energy consumption of the process also has an impact on the total water migration amount.

### 3.3. Preliminary Economic Analysis

To assess the feasibility of using ED to concentrate LiCl and its potential for industrial application, an economic evaluation is essential. The economic assessment primarily focuses on two components: capital investment costs and energy consumption costs. Capital investment costs include the costs of membranes, membrane stacks, and equipment. On the other hand, energy consumption costs include the energy consumption of membrane stacks used in ED and the energy consumption of equipment. Based on existing economic evaluation methods [48,49] the optimal concentration conditions obtained from scaling-up experiment were selected, and the economic assessment results are shown in Table 3.

The economic evaluation results indicate that the total energy cost of the two-level ED process used in this study to concentrate LiCl is 8.52 USD/t LiCl, which saves 14.66 USD/t LiCl compared to the traditional evaporation method. Most of the costs are fixed investment costs, and the high cost of ion exchange membranes is one of the main factors contributing to this result. Therefore, exploring methods for reducing membrane costs, minimizing membrane fouling losses, and extending membrane lifespan are crucial for promoting the industrial application of ED. Thus, the multi-level ED process for LiCl concentration is economically feasible and has significant industrialization potential.

## 4. Conclusions

This study has successfully demonstrated the technical and economic viability of a two-level ED process for industrial-scale LiCl concentration. Through systematic optimization of operational parameters, the process achieved remarkable performance metrics of a Li^+^ concentration of 22.17 g/L in the final concentrate solution and 21.17 g/L in the recycled dilute solution, while maintaining discharge water concentrations at just 1.08 g/L—well below environmental compliance thresholds. The energy efficiency of the system was particularly noteworthy, with total specific energy consumption measured at 85.22 kWh/t LiCl, representing a 63% reduction compared to conventional evaporation methods (231.8 kWh/t LiCl). Water management was significantly improved, with the optimized process limiting total water migration amount to 4.21 L/(m^2^·h), a substantial improvement over initial small-scale experiments that showed water migration amounts as high as 24.69 L/(m^2^·h). Economic analysis revealed compelling cost advantages, with the ED process delivering savings of 14.66 USD/t LiCl compared to traditional thermal concentration methods. These results were achieved under the following carefully controlled conditions: the constant voltage setting for the experiment was 60V, temperature range was 29–31 °C, and the specific volume ratios were 20:3 for the concentrate solution and 27:3 for the dilute solution in the second-level concentration process. The study’s findings provide a robust foundation for industrial implementation, though further research could enhance commercial viability through the development of lower-cost ion exchange membranes (currently accounting for approximately 60% of capital costs) and the optimization of membrane lifespan under continuous operation. The demonstrated process represents a significant advancement in sustainable lithium processing technology, offering both environmental benefits through reduced energy and water usage, and economic advantages through lower operating costs, while meeting the increasing global demand for high-purity lithium compounds in energy storage applications.

## Figures and Tables

**Table 1:** Key parameters of the LiCl raw material solution.

Parameters	Mass Fraction (%)	pH	Conductivity (mS/cm)
LiCl raw material solution	7.68	7.06	75.6

**Table 2:** Key parameters of the ion exchange membrane.

Membrane	Thickness (μm)	Surface Area Resistance (Ω·cm^2^)	Ion-Exchange Capacity (meq/g)	Temperature (°C)
CMT	120–130	3 ^a^	1.1	≤35
AMT	120–130	5 ^b^	0.8	≤35

^a^ The area resistance of ion exchange membrane was measured with 25 °C and a 1 M NaCl solution. ^b^ The area resistance of ion exchange membrane was measured with 25 °C and a 1 M NaCl solution.

**Table 3:** Economic evaluation table for LiCl concentration by ED.

Operating Conditions	
Number of repeating units	60
Actual area of single membrane (m^2^)	0.125
Total effective area of membrane (m^2^)	4.275
Flow rate of each chamber (m^3^/h)	1
Feed conductivity (mS/cm)	75.3
Voltage (V)	60
fixed investment costs	
CMT cation exchange membrane (USD/m^2^)	120
AMT anion exchange membrane (USD/m^2^)	120
Membrane cost [48,49] (USD)	1815
Membrane stack cost (USD) ^a^	2722.5
Equipment cost (USD) ^b^	4083.75
Total fixed investment cost (USD/year) ^c^	1317.3
Energy consumption costs	
ED energy consumption(kWh/t LiCl)	85.22
Equipment energy consumption (kWh/t LiCl)	22.97
Electricity cost (USD/kWh)	0.1
Energy consumption cost (USD/t LiCl)	8.52

^a^ The cost of the membrane stack is calculated as 1.5 times the cost of the membrane. ^b^ The cost of the equipment is calculated as 1.5 times the cost of the membrane stack. ^c^ The service life of the membrane is calculated as 3 years, and the service life of the equipment is calculated as 10 years.

## Data Availability

The original contributions presented in this study are included in the article. Further inquiries can be directed to the corresponding author.

## Nomenclature

CEM	Cation exchange membrane
AEM	Anion exchange membrane
CMT	Cation exchange membrane model
AMT	Anion exchange membrane model
MVR	Mechanical vapor recompression
ED	Electrodialysis
CC	Concentrate chamber
DC	Dilute chamber
